# Supplementary material for: Facial emotion recognition abilities of individuals with schizophrenia and the influence of parental bonding—An exploratory study in a forensic sample
Source: PLoS One. 2026 Feb 10;21(2):e0339713. doi: 10.1371/journal.pone.0339713 (PMC12890136; doi:10.1371/journal.pone.0339713)
Supplement: S1 Table — (DOCX) [file pone.0339713.s001.docx]

**Supplementary Table 1**: Analysis of Deviance Table for the GLM tested against the grand mean of FER an error rate.

| Effect | SumSq | *Df* | *F* | *p* | ω_p_^2^ |
| --- | --- | --- | --- | --- | --- |
| *Group* | 0.179 | 1 | 19.02 | <.0001 | 0.28 |
| *parenting style* | 0.038 | 1 | 4.07 | 0.049 | 0.06 |
| *group* × *parenting style* | 0.050 | 1 | 5.33 | 0.03 | 0.10 |
| Residuals | 0.432 | 46 |  |  |  |
