# Supplementary material for: Facial emotion recognition abilities of individuals with schizophrenia and the influence of parental bonding—An exploratory study in a forensic sample
Source: PLoS One. 2026 Feb 10;21(2):e0339713. doi: 10.1371/journal.pone.0339713 (PMC12890136; doi:10.1371/journal.pone.0339713)
Supplement: S2 Table — CL: 95% confidence limits for means. (DOCX) [file pone.0339713.s002.docx]

**Supplementary Table 2**: Estimated marginal means of FER error rate in both groups under both parenting styles. CL: 95 % confidence limits for means.

| Group | Parenting Style | Error Rate | SE | lowerCL | upperCL |
| --- | --- | --- | --- | --- | --- |
| CTL | optimal parenting | 0.263 | 0.023 | 0.219 | 0.308 |
| CTL | neglectful parenting | 0.257 | 0.031 | 0.197 | 0.317 |
| PAT | optimal parenting | 0.319 | 0.029 | 0.262 | 0.377 |
| PAT | neglectful parenting | 0.455 | 0.029 | 0.397 | 0.512 |
