# Supplementary material for: Facial emotion recognition abilities of individuals with schizophrenia and the influence of parental bonding—An exploratory study in a forensic sample
Source: PLoS One. 2026 Feb 10;21(2):e0339713. doi: 10.1371/journal.pone.0339713 (PMC12890136; doi:10.1371/journal.pone.0339713)
Supplement: S3 Table — (DOCX) [file pone.0339713.s003.docx]

**Supplementary Table 3**: Simple main effect contrast estimates.

| Contrast | Estimate | SE | lower CL | upper CL | *t* | *p* | Hedges‘ g |
| --- | --- | --- | --- | --- | --- | --- | --- |
| CTL optimal - PAT optimal | -0.056 | 0.0371 | -0.151 | 0.039 | -1.509 | .14 | 0.56 |
| CTL optimal - CTL neglectful | 0.006 | 0.0382 | -0.092 | 0.105 | 0.166 | .87 | 0.06 |
| PAT optimal - PAT neglectful | -0.135 | 0.0413 | -0.241 | -0.029 | -3.267 | .002 | 1.34 |
| CTL neglectful - PAT neglectful | -0.197 | 0.0424 | -0.306 | -0.089 | -4.660 | <.001 | 1.96 |

Note. all *DF* = 46
