# Supplementary material for: Facial emotion recognition abilities of individuals with schizophrenia and the influence of parental bonding—An exploratory study in a forensic sample
Source: PLoS One. 2026 Feb 10;21(2):e0339713. doi: 10.1371/journal.pone.0339713 (PMC12890136; doi:10.1371/journal.pone.0339713)
Supplement: S4 Table — (DOCX) [file pone.0339713.s004.docx]

**Supplementary Table 4**: Spearman’s ρ correlation coefficients between the PBI scores and FERerr.

| **CTL & PAT pooled** | **ρ** | ***p*** |
| --- | --- | --- |
| PBI Mother Care | 0.22 | 0.23 |
| PBI Mother Overprotection | -0.19 | 0.30 |
| PBI Father Care | 0.07 | 0.70 |
| PBI Father Overprotection | -0.25 | 0.17 |
|  |  |  |
| **CTL** | **ρ** | ***p*** |
| PBI Mother Care | 0.10 | 0.72 |
| PBI Mother Overprotection | 0.12 | 0.65 |
| PBI Father Care | -0.15 | 0.58 |
| PBI Father Overprotection | 0.21 | 0.45 |
|  |  |  |
| **PAT** | **ρ** | ***p*** |
| PBI Mother Care | 0.10 | 0.71 |
| PBI Mother Overprotection | -0.32 | 0.23 |
| PBI Father Care | 0.36 | 0.21 |
| PBI Father Overprotection | -0.25 | 0.38 |
