# Supplementary material for: Facial emotion recognition abilities of individuals with schizophrenia and the influence of parental bonding—An exploratory study in a forensic sample
Source: PLoS One. 2026 Feb 10;21(2):e0339713. doi: 10.1371/journal.pone.0339713 (PMC12890136; doi:10.1371/journal.pone.0339713)
Supplement: S5 Table — (DOCX) [file pone.0339713.s005.docx]

**Supplementary Table 5:** A comparison of recent studies of facial recognition (FER) impairment in schizophrenia and control groups.

| **Group/Condition** | **Key FER Deficits** | **Emotions Most Affected** | **Response Time** | **Notable Findings** | **Citations** |
| --- | --- | --- | --- | --- | --- |
| Schizophrenia (general) | Lower accuracy, more errors | Fear, anger, sadness, contempt | Slower | Deficits in both accuracy and speed; errors include mislabeling positive as negative emotions | (Bae et al., 2024; Lee et al., 2021; Pena-Garijo et al., 2022; Hachtel et al., 2024; Kjellenberg & Winblad, 2020; Kuang et al., 2022; Kuang et al., 2021) |
| First-episode schizophrenia | Impaired accuracy | Fear (most), happy | Slower | Fear recognition especially impaired; linked to brain activity changes | (Kuang et al., 2022; Kuang et al., 2021) |
| Multi-episode schizophrenia | Persistent deficits | Fear, anger | Slower | Deficits increase with illness duration | (Pena-Garijo et al., 2022) |
| High-risk/First-degree relatives | Mild to moderate deficits | Fear, contempt | Slightly slower | Some deficits present, especially for fear and contempt | (Bae et al., 2024; Pena-Garijo et al., 2022; Fusar-Poli et al., 2021) |
| Healthy controls | Baseline (best performance) | None | Fastest | Serve as reference group | (Bae et al., 2024; Lee et al., 2021; Pena-Garijo et al., 2022; Fusar-Poli et al., 2021; Kuang et al., 2022; Kjellenberg & Winblad, 2020; Kuang et al., 2022; Kuang et al., 2021) |
| Other psychotic disorders | Less severe than schizophrenia | Anger (some), fear | Slower than controls | Not always significantly different from controls | (Kjellenberg & Winblad, 2020) |

**References**

Bae, M., Cho, J., & Won, S. (2024). Facial emotion-recognition deficits in patients with schizophrenia and unaffected first-degree relatives. *Frontiers in Psychiatry*, 15. <https://doi.org/10.3389/fpsyt.2024.1373288>

Lee, S., Lin, G., Shih, C., Chen, K., Liu, C., Kuo, C., & Hsieh, C. (2021). Error patterns of facial emotion recognition in patients with schizophrenia.. *Journal of affective disorders*. <https://doi.org/10.1016/j.jad.2021.12.130>

Pena-Garijo, J., Lacruz, M., Masanet, M., Palop-Grau, A., Plaza, R., Hernández-Merino, A., Edo-Villamón, S., & Valllina, O. (2022). Specific facial emotion recognition deficits across the course of psychosis: A comparison of individuals with low-risk, high-risk, first-episode psychosis and multi-episode schizophrenia-spectrum disorders. *Psychiatry Research*, 320. <https://doi.org/10.1016/j.psychres.2022.115029>

Fusar-Poli, L., Pries, L., Os, J., Erzin, G., Delespaul, P., Kenis, G., Luykx, J., Lin, B., Richards, A., Akdede, B., Binbay, T., Altınyazar, V., Yalınçetin, B., Gümüş-Akay, G., Cihan, B., Soygür, H., Ulaş, H., Cankurtaran, E., Kaymak, S., Mihaljevic, M., Andrić-Petrović, S., Mirjanić, T., Bernardo, M., Mezquida, G., Amoretti, S., Bobes, J., Sáiz, P., García-Portilla, M., Sanjuán, J., Aguilar, E., Santos, J., Jiménez-López, E., Arrojo, M., Carracedo, Á., López, G., González-Peñas, J., Parellada, M., Maric, N., Atbaşoğlu, C., Üçok, A., Alptekin, K., Saka, M., Aguglia, E., Arango, C., O’Donovan, M., Rutten, B., & Guloksuz, S. (2021). Examining facial emotion recognition as an intermediate phenotype for psychosis: Findings from the EUGEI study. *Progress in Neuro-Psychopharmacology and Biological Psychiatry*, 113. <https://doi.org/10.1016/j.pnpbp.2021.110440>

Kuang, Q., Zhou, S., Liu, Y., Wu, H., Bi, T., She, S., & Zheng, Y. (2022). Prediction of Facial Emotion Recognition Ability in Patients With First-Episode Schizophrenia Using Amplitude of Low-Frequency Fluctuation-Based Support Vector Regression Model. *Frontiers in Psychiatry*, 13. <https://doi.org/10.3389/fpsyt.2022.905246>

Fusar-Poli, L., Pries, L., Van Os, J., Radhakrishnan, R., Pence, A., Erzin, G., Delespaul, P., Kenis, G., Luykx, J., Lin, B., Akdede, B., Binbay, T., Altınyazar, V., Yalınçetin, B., Gümüş-Akay, G., Cihan, B., Soygür, H., Ulaş, H., Cankurtaran, E., Kaymak, S., Mihaljevic, M., Andrić-Petrović, S., Mirjanić, T., Bernardo, M., Mezquida, G., Amoretti, S., Bobes, J., Sáiz, P., García-Portilla, M., Sanjuán, J., Aguilar, E., Santos, J., Jiménez-López, E., Arrojo, M., Carracedo, Á., López, G., González-Peñas, J., Parellada, M., Maric, N., Atbaşoğlu, C., Üçok, A., Alptekin, K., Saka, M., Aguglia, E., Arango, C., Rutten, B., & Guloksuz, S. (2022). The association between cannabis use and facial emotion recognition in schizophrenia, siblings, and healthy controls: Results from the EUGEI study. *European Neuropsychopharmacology*, 63, 47-59. <https://doi.org/10.1016/j.euroneuro.2022.08.003>

Hachtel, H., Deuring, G., Graf, M., & Vogel, T. (2024). Impact of psychosocial stress on facial emotion recognition in schizophrenia and controls: an experimental study in a forensic sample. *Frontiers in Psychiatry*, 15. <https://doi.org/10.3389/fpsyt.2024.1358291>

Kjellenberg, E., & Winblad, S. (2020). M74. FACIAL EMOTION RECOGNITION ABILITY IN PATIENTS WITH SCHIZOPHRENIA AND OTHER PSYCHOTIC DISORDERS. *Schizophrenia Bulletin*, 46, S163 - S163. <https://doi.org/10.1093/schbul/sbaa030.386>

Kuang, Q., Zhou, S., Li, H., Mi, L., Zheng, Y., & She, S. (2022). Association between fractional amplitude of low-frequency fluctuation (fALFF) and facial emotion recognition ability in first-episode schizophrenia patients: a fMRI study. *Scientific Reports*, 12. <https://doi.org/10.1038/s41598-022-24258-7>

Kuang, Q., Liu, Y., Zhou, S., Bi, T., Mi, L., She, S., & Zheng, Y. (2021). The correlation between fractional amplitude of low-frequency fluctuation-based resting-state functional magnetic resonance imaging and facial emotion recognition ability in patients with first-episode schizophrenia. **. <https://doi.org/10.21203/rs.3.rs-919680/v1>
