# Supplementary material for: Facial emotion recognition abilities of individuals with schizophrenia and the influence of parental bonding—An exploratory study in a forensic sample
Source: PLoS One. 2026 Feb 10;21(2):e0339713. doi: 10.1371/journal.pone.0339713 (PMC12890136; doi:10.1371/journal.pone.0339713)
Supplement: S1 File — (DOCX) [file pone.0339713.s006.docx]

**List of Karolinska Directed Emotional Faces images used in FER A.**

AF01AFS, AF01ANS, AF01DIS, AF01HAS, AF01SAS, AF02AFS, AF02ANS, AF02DIS, AF02HAS, AF02SAS, AF03AFS, AF03ANS, AF03DIS, AF03HAS, AF03SAS, AM01AFS, AM01ANS, AM01DIS, AM01HAS, AM01SAS, AM02AFS, AM02ANS, AM02DIS, AM02HAS, AM02SAS, AM04AFS, AM04ANS, AM04DIS, AM04HAS, AM04SAS, AM05AFS, AM05ANS, AM05DIS, AM05HAS, AM05SAS
