# Supplementary material for: Facial emotion recognition abilities of individuals with schizophrenia and the influence of parental bonding—An exploratory study in a forensic sample
Source: PLoS One. 2026 Feb 10;21(2):e0339713. doi: 10.1371/journal.pone.0339713 (PMC12890136; doi:10.1371/journal.pone.0339713)
Supplement: S2 File — (DOCX) [file pone.0339713.s007.docx]

## **Statistical Analysis Pipeline of the GLM in R (core components)**

require(emmeans)

require(car)

# Generalized linear model (GLM) with square root link function

mod = glm(err.rate ~ group * par.style , data=pbi.dat4, family = gaussian(link="sqrt"))

# Analysis of Deviance Table (Type III tests) of model effects

car::Anova(mod, type=3, test="F")

effectsize::omega_squared(mod, ci=0.9)

# Backtransformed estimated marginal means and post hoc contrasts

emms = emmeans::emmeans(mod, ~ group * par.style)

summary(emmeans::regrid(emms))

# backtransformed contrasts, no adjustment

emmeans::emm_options(contrast = list(infer = c(TRUE, TRUE), adjust="none"))

emmeans::contrast(emmeans::regrid(emms), method = "pairwise", adjust="none", level=0.95)

# Not mentioned in the manuscript:
# Bootstrapping based parameter and confidence interval estimation

require(boot)

lm_coef = function(dat, inds){
 lm.b = glm(err.rate ~ group * par.style , data=dat[inds,], family = gaussian(link="sqrt"))
 coef(lm.b)
}

boot_results = boot::boot(data=pbi.dat4, statistic=lm_coef, R=10000)

boot_ci = lapply(c(1:4), function(x) as.data.frame(t(boot::boot.ci(boot_results, type = "perc", index = x)$percent[4:5])))

boot_ci = do.call(rbind, boot_ci)

names(boot_ci) = c("bootCI_lower", "bootCI_upper")

boot_ci = data.frame(effect=c("Intercept", "group","par.style","group:par.style"), boot_ci)

# post hoc emmeans

ph_emm = function(dat, inds){
 lm.b = glm(err.rate ~ group * par.style , data=dat[inds,], family = gaussian(link="sqrt"))
 results = as.data.frame(emmeans(lm.b, ~ group * par.style, type= "response", adjust="none"))
 unlist(results$response)
}

boot_ph = boot(data=pbi.dat4, statistic=ph_emm, R=10000)

summary(boot_ph)

boot_ci_ph = lapply(c(1:4), function(x) as.data.frame(t(boot.ci(boot_ph, type = "perc", index = x)$percent[4:5])))

boot_ci_ph = do.call(rbind, boot_ci_ph)

names(boot_ci_ph) = c("bootCI_lower", "bootCI_upper")

ph_names = as.data.frame(emmeans(mod, ~ group * par.style, type= "response", adjust="none"))[,1:2]

boot_ci_ph = data.frame(ph_names, as.data.frame(summary(boot_ph)), boot_ci_ph)
