# Supplementary material for: Facial emotion recognition abilities of individuals with schizophrenia and the influence of parental bonding—An exploratory study in a forensic sample
Source: PLoS One. 2026 Feb 10;21(2):e0339713. doi: 10.1371/journal.pone.0339713 (PMC12890136; doi:10.1371/journal.pone.0339713)
Supplement: Attachment — Submitted filename: Response to Reviewer Comments.DOC [file pone.0339713.s009.DOC]

Response to Reviewer Comments

We would like to thank the reviewers for their valuable and insightful reviews and comments on our work. We are grateful for the opportunity to improve our manuscript. We believe that the revisions we have made have substantially strengthened the paper, and we look forward to your decision.

We have revised and supplemented the manuscript accordingly, particularly in the Introduction, Materials and Methods, Discussion, and Conclusion/Outlook sections.

Below, we have provided detailed answers to the respective questions.

**Journal Requirements:**

When submitting your revision, we need you to address these additional requirements.

1. Please ensure that your manuscript meets PLOS ONE's style requirements, including those for file naming. The PLOS ONE style templates can be found at

[https://journals.plos.org/plosone/s/file?id=wjVg/PLOSOne_formatting_sample_main_body.pdf](https://urldefense.com/v3/__https:/journals.plos.org/plosone/s/file?id=wjVg*PLOSOne_formatting_sample_main_body.pdf__;Lw!!EDSXHx-qqdzzoNk!vpIivTUAqJlYhBM6h24PPHLTnhbYPAbGXwb8U3iWL-i7ftAfsAkxFVZP_qgzb3-JP86mrOUrx8aXVBrkUIs-JER9sw$) and [https://journals.plos.org/plosone/s/file?id=ba62/PLOSOne_formatting_sample_title_authors_affiliations.pdf](https://urldefense.com/v3/__https:/track.editorialmanager.com/CL0/https:*2F*2Fjournals.plos.org*2Fplosone*2Fs*2Ffile*3Fid=ba62*2FPLOSOne_formatting_sample_title_authors_affiliations.pdf/1/010f0198333da4a1-681f2696-281b-4281-8fc5-b571a5df4dc7-000000/8zQICY94Ee8KQF2Ry8VI4ul0R4To8sZBfU50xPg5gAA=220__;JSUlJSUlJQ!!EDSXHx-qqdzzoNk!vpIivTUAqJlYhBM6h24PPHLTnhbYPAbGXwb8U3iWL-i7ftAfsAkxFVZP_qgzb3-JP86mrOUrx8aXVBrkUIsnWOJCmg$)

*Answer: The style guidelines have been implemented.*

2. Please describe in your methods section how capacity to provide consent was determined for the participants in this study. Please also state whether your ethics committee or IRB approved this consent procedure. If you did not assess capacity to consent please briefly outline why this was not necessary in this case.

*Answer: The capacity to consent was assessed and confirmed by the treating physicians for participating patients.*

*The information was added to the manuscript.*

3. Thank you for stating in your Funding Statement:

[This work was supported by the Forschungsförderungsfonds of the University Psychiatric Clinics of Basel. The authors declare financial support was received for the research.].

Please provide an amended statement that declares *all* the funding or sources of support (whether external or internal to your organization) received during this study, as detailed online in our guide for authors at [http://journals.plos.org/plosone/s/submit-now](https://urldefense.com/v3/__http:/journals.plos.org/plosone/s/submit-now__;!!EDSXHx-qqdzzoNk!vpIivTUAqJlYhBM6h24PPHLTnhbYPAbGXwb8U3iWL-i7ftAfsAkxFVZP_qgzb3-JP86mrOUrx8aXVBrkUIvZLqAn9A$).  Please also include the statement “There was no additional external funding received for this study.” in your updated Funding Statement.

Please include your amended Funding Statement within your cover letter. We will change the online submission form on your behalf.

*Answer: This work was financially supported by the “Forschungsförderungsfonds” of the University Psychiatric Clinics of Basel only. There were no other funds or sources of financial support involved. This information was added to the cover letter.*

4. We note that Figure 1 includes an image of a patient/participant in the study.

As per the PLOS ONE policy ([http://journals.plos.org/plosone/s/submission-guidelines#loc-human-subjects-research](https://urldefense.com/v3/__http:/journals.plos.org/plosone/s/submission-guidelines*loc-human-subjects-research__;Iw!!EDSXHx-qqdzzoNk!vpIivTUAqJlYhBM6h24PPHLTnhbYPAbGXwb8U3iWL-i7ftAfsAkxFVZP_qgzb3-JP86mrOUrx8aXVBrkUIs_o1eVVA$)) on papers that include identifying, or potentially identifying, information, the individual(s) or parent(s)/guardian(s) must be informed of the terms of the PLOS open-access (CC-BY) license and provide specific permission for publication of these details under the terms of this license. Please download the Consent Form for Publication in a PLOS Journal ([http://journals.plos.org/plosone/s/file?id=8ce6/plos-consent-form-english.pdf](https://urldefense.com/v3/__http:/journals.plos.org/plosone/s/file?id=8ce6*plos-consent-form-english.pdf__;Lw!!EDSXHx-qqdzzoNk!vpIivTUAqJlYhBM6h24PPHLTnhbYPAbGXwb8U3iWL-i7ftAfsAkxFVZP_qgzb3-JP86mrOUrx8aXVBrkUIteM5tI1Q$)). The signed consent form should not be submitted with the manuscript, but should be securely filed in the individual's case notes. Please amend the methods section and ethics statement of the manuscript to explicitly state that the patient/participant has provided consent for publication: “The individual in this manuscript has given written informed consent (as outlined in PLOS consent form) to publish these case details”.

If you are unable to obtain consent from the subject of the photograph, you will need to remove the figure and any other textual identifying information or case descriptions for this individual.

*Answer: The depiction of a human face in Figure 1 is a stimulus picture of the FER test and does not represent a participant in the study.*

*The image of a human face in Figure 1 is a sample stimulus image as it was used in the Facial Emotion Recognition task A (FER A). The images are taken from the Karolinska Directed Emotional Faces picture set (KDEF; Lundquist, Flykt, & Öhman, 1998) with the full permission for use in research and publication. The ID of the specific image in Figure 1 is AF01SAS.*

*For the KDEF terms and conditions, see here:*

[*https://kdef.se/faq/using-and-publishing-kdef-and-akdef*](https://urldefense.com/v3/__https:/kdef.se/faq/using-and-publishing-kdef-and-akdef__;!!EDSXHx-qqdzzoNk!rI4hITtYylVRI7g6-oPyW41clyrk1AvXElvahQyQ9MbtVEW6ISv731-t19cn-MD-QTp8La5DNIBPM7U3REmW1sIYxOQH$)

*The origin of the stimulus images is stated in the description of the FER A in the Methods section of the manuscript. We noticed that the term used there, “Karolinska standard”, is not entirely correct, so we replaced it by the correct official name “Karolinska Directed Emotional Faces”.*

*Upon reviewing the KDEF terms and conditions, we also became aware that the KDEF publishers wish a list of all stimulus images used in the experimental paradigm to be published alongside the manuscript, so we added the full list of image IDs to the Supplementary Material and added a reference to the manuscript “A list of all stimulus image IDs used in the FER A paradigm can be found in the Supplementary Material”.*

*We further added the following statement to the caption of Figure 1: “The emotional face stimulus image is ID AF01SAS from the Karolinska Directed Emotional Faces set.*

5. Please include captions for your Supporting Information files at the end of your manuscript, and update any in-text citations to match accordingly. Please see our Supporting Information guidelines for more information: [http://journals.plos.org/plosone/s/supporting-information](https://urldefense.com/v3/__http:/journals.plos.org/plosone/s/supporting-information__;!!EDSXHx-qqdzzoNk!vpIivTUAqJlYhBM6h24PPHLTnhbYPAbGXwb8U3iWL-i7ftAfsAkxFVZP_qgzb3-JP86mrOUrx8aXVBrkUIupYm8s1Q$).

*Answer: The citations have been updated.*

Review Comments to the Author

Please use the space provided to explain your answers to the questions above. You may also include additional comments for the author, including concerns about dual publication, research ethics, or publication ethics. (Please upload your review as an attachment if it exceeds 20,000 characters)

Reviewer #1:

I have reviewed the manuscript titled “Facial Emotion Recognition Abilities of Individuals with Schizophrenia and the Influence of Parental Bonding – An Exploratory Study in a Forensic Sample.” The study addresses an important and underexplored area, investigating how parental bonding patterns influence facial emotion recognition (FER) performance in individuals with schizophrenia within a forensic sample. The findings are clinically relevant, as they suggest that optimal parental attachment may mitigate FER deficits, while neglectful parenting is associated with poorer performance. To further strengthen the study, I suggest the authors consider sharing their full analysis code and data processing pipeline (where ethically possible) to promote reproducibility.

*Answer: The R packets used for the analysis and the description given in the manuscript should be sufficient for anybody seeking to reproduce the generalized linear model (GLM) as it was applied here. Nonetheless, we added the core analysis commands to the Supplementary Material.*

*Study data in a forensic setting is very sensitive and protected more strictly by law and cannot be shared easily with the public.*

-Including a more detailed subgroup or ablation analysis could help disentangle the specific contributions of different parental bonding dimensions (care vs. overprotection) to FER outcomes.

*Answer: The sample size, respectively, the cell fillings resulting from the PBI classification is already at the limit of minimum statistical requirements and definitely too small to conduct subgroup analyses. The presented work is merely a secondary analysis of pre-existing data, however, a future study on parenting style and FER would be powered accordingly and allow for more sophisticated analyses. We are not familiar with the term “ablation analysis”.*

- Validation on independent or more diverse cohorts would also help confirm the robustness of the findings. The discussion would benefit from a state of the art comparison table summarizing existing FER studies in schizophrenia to better contextualize the current results.

Deficits in facial emotion recognition (FER) are a well-established feature of schizophrenia, affecting both accuracy and response time. These deficits are present across different stages of the condition and in different risk groups. FER deficits in schizophrenia are consistent, particularly for negative emotions such as fear and anger, and are evident from the early stages of the condition and in individuals at high risk of developing it. These deficits are more pronounced and persistent in schizophrenia than in other psychotic disorders or in individuals without mental health conditions.

*Answer: In addition to the studies already mentioned in the manuscript, we have summarized the current state of research on FER in schizophrenia in a table.*

*A comparison of some recent studies of facial emotion recognition (FER) impairment* across schizophrenia risk groups and control groups:

| **Group/Condition** | **Key FER Deficits** | **Emotions Most Affected** | **Response Time** | **Notable Findings** | **Citations** |
| --- | --- | --- | --- | --- | --- |
| Schizophrenia (general) | Lower accuracy, more errors | Fear, anger, sadness, contempt | Slower | Deficits in both accuracy and speed; errors include mislabeling positive as negative emotions | (Bae et al., 2024; Lee et al., 2021; Pena-Garijo et al., 2022; Hachtel et al., 2024; Kjellenberg & Winblad, 2020; Kuang et al., 2022; Kuang et al., 2021) |
| First-episode schizophrenia | Impaired accuracy | Fear (most), happy | Slower | Fear recognition especially impaired; linked to brain activity changes | (Kuang et al., 2022; Kuang et al., 2021) |
| Multi-episode schizophrenia | Persistent deficits | Fear, anger | Slower | Deficits increase with illness duration | (Pena-Garijo et al., 2022) |
| High-risk/First-degree relatives | Mild to moderate deficits | Fear, contempt | Slightly slower | Some deficits present, especially for fear and contempt | (Bae et al., 2024; Pena-Garijo et al., 2022; Fusar-Poli et al., 2021) |
| Healthy controls | Baseline (best performance) | None | Fastest | Serve as reference group | (Bae et al., 2024; Lee et al., 2021; Pena-Garijo et al., 2022; Fusar-Poli et al., 2021; Kuang et al., 2022; Kjellenberg & Winblad, 2020; Kuang et al., 2022; Kuang et al., 2021) |
| Other psychotic disorders | Less severe than schizophrenia | Anger (some), fear | Slower than controls | Not always significantly different from controls | (Kjellenberg & Winblad, 2020) |

***References***

1. *Bae, M., Cho, J., & Won, S. (2024). Facial emotion-recognition deficits in patients with schizophrenia and unaffected first-degree relatives. Frontiers in Psychiatry, 15.* [*https://doi.org/10.3389/fpsyt.2024.1373288*](https://doi.org/10.3389/fpsyt.2024.1373288)
2. *Lee, S., Lin, G., Shih, C., Chen, K., Liu, C., Kuo, C., & Hsieh, C. (2021). Error patterns of facial emotion recognition in patients with schizophrenia.. Journal of affective disorders.* [*https://doi.org/10.1016/j.jad.2021.12.130*](https://doi.org/10.1016/j.jad.2021.12.130)
3. *Pena-Garijo, J., Lacruz, M., Masanet, M., Palop-Grau, A., Plaza, R., Hernández-Merino, A., Edo-Villamón, S., & Valllina, O. (2022). Specific facial emotion recognition deficits across the course of psychosis: A comparison of individuals with low-risk, high-risk, first-episode psychosis and multi-episode schizophrenia-spectrum disorders. Psychiatry Research, 320.* [*https://doi.org/10.1016/j.psychres.2022.115029*](https://doi.org/10.1016/j.psychres.2022.115029)
4. *Fusar-Poli, L., Pries, L., Os, J., Erzin, G., Delespaul, P., Kenis, G., Luykx, J., Lin, B., Richards, A., Akdede, B., Binbay, T., Altınyazar, V., Yalınçetin, B., Gümüş-Akay, G., Cihan, B., Soygür, H., Ulaş, H., Cankurtaran, E., Kaymak, S., Mihaljevic, M., Andrić-Petrović, S., Mirjanić, T., Bernardo, M., Mezquida, G., Amoretti, S., Bobes, J., Sáiz, P., García-Portilla, M., Sanjuán, J., Aguilar, E., Santos, J., Jiménez-López, E., Arrojo, M., Carracedo, Á., López, G., González-Peñas, J., Parellada, M., Maric, N., Atbaşoğlu, C., Üçok, A., Alptekin, K., Saka, M., Aguglia, E., Arango, C., O’Donovan, M., Rutten, B., & Guloksuz, S. (2021). Examining facial emotion recognition as an intermediate phenotype for psychosis: Findings from the EUGEI study. Progress in Neuro-Psychopharmacology and Biological Psychiatry, 113.* [*https://doi.org/10.1016/j.pnpbp.2021.110440*](https://doi.org/10.1016/j.pnpbp.2021.110440)
5. *Kuang, Q., Zhou, S., Liu, Y., Wu, H., Bi, T., She, S., & Zheng, Y. (2022). Prediction of Facial Emotion Recognition Ability in Patients With First-Episode Schizophrenia Using Amplitude of Low-Frequency Fluctuation-Based Support Vector Regression Model. Frontiers in Psychiatry, 13.* [*https://doi.org/10.3389/fpsyt.2022.905246*](https://doi.org/10.3389/fpsyt.2022.905246)
6. *Fusar-Poli, L., Pries, L., Van Os, J., Radhakrishnan, R., Pence, A., Erzin, G., Delespaul, P., Kenis, G., Luykx, J., Lin, B., Akdede, B., Binbay, T., Altınyazar, V., Yalınçetin, B., Gümüş-Akay, G., Cihan, B., Soygür, H., Ulaş, H., Cankurtaran, E., Kaymak, S., Mihaljevic, M., Andrić-Petrović, S., Mirjanić, T., Bernardo, M., Mezquida, G., Amoretti, S., Bobes, J., Sáiz, P., García-Portilla, M., Sanjuán, J., Aguilar, E., Santos, J., Jiménez-López, E., Arrojo, M., Carracedo, Á., López, G., González-Peñas, J., Parellada, M., Maric, N., Atbaşoğlu, C., Üçok, A., Alptekin, K., Saka, M., Aguglia, E., Arango, C., Rutten, B., & Guloksuz, S. (2022). The association between cannabis use and facial emotion recognition in schizophrenia, siblings, and healthy controls: Results from the EUGEI study. European Neuropsychopharmacology, 63, 47-59.* [*https://doi.org/10.1016/j.euroneuro.2022.08.003*](https://doi.org/10.1016/j.euroneuro.2022.08.003)
7. *Hachtel, H., Deuring, G., Graf, M., & Vogel, T. (2024). Impact of psychosocial stress on facial emotion recognition in schizophrenia and controls: an experimental study in a forensic sample. Frontiers in Psychiatry, 15.* [*https://doi.org/10.3389/fpsyt.2024.1358291*](https://doi.org/10.3389/fpsyt.2024.1358291)
8. *Kjellenberg, E., & Winblad, S. (2020). M74. FACIAL EMOTION RECOGNITION ABILITY IN PATIENTS WITH SCHIZOPHRENIA AND OTHER PSYCHOTIC DISORDERS. Schizophrenia Bulletin, 46, S163 - S163.* [*https://doi.org/10.1093/schbul/sbaa030.386*](https://doi.org/10.1093/schbul/sbaa030.386)
9. *Kuang, Q., Zhou, S., Li, H., Mi, L., Zheng, Y., & She, S. (2022). Association between fractional amplitude of low-frequency fluctuation (fALFF) and facial emotion recognition ability in first-episode schizophrenia patients: a fMRI study. Scientific Reports, 12.* [*https://doi.org/10.1038/s41598-022-24258-7*](https://doi.org/10.1038/s41598-022-24258-7)
10. *Kuang, Q., Liu, Y., Zhou, S., Bi, T., Mi, L., She, S., & Zheng, Y. (2021). The correlation between fractional amplitude of low-frequency fluctuation-based resting-state functional magnetic resonance imaging and facial emotion recognition ability in patients with first-episode schizophrenia. **.* [*https://doi.org/10.21203/rs.3.rs-919680/v1*](https://doi.org/10.21203/rs.3.rs-919680/v1)

- A dedicated limitations and future work section should be expanded to explicitly address issues such as sample size, gender restriction, retrospective bias in PBI reporting, and generalizability. Finally, the inclusion and exclusion criteria for participant selection should be clearly summarized for clarity.

*Answer: In the section on strengths and weaknesses and discussion, the relevant information on sample size, gender restriction, retrospective bias in PBI reporting, and generalisability was added. The inclusion and exclusion criteria were outlined more clearly in the introduction.*

- For further methodological inspiration, particularly on advanced feature extraction and classification in psychiatric populations, the authors may consider reviewing the following works: “Zipper Pattern: An Investigation into Psychotic Criminal Detection Using EEG Signals,” “Multilevel Hybrid Handcrafted Feature Extraction Based Depression Recognition Method Using Speech,” and “Automated Schizophrenia Detection Model Using Blood Sample Scattergram Images and Local Binary Pattern.” These studies illustrate how explainable and structured computational models can enhance the understanding and classification of complex psychiatric and neurocognitive phenomena offering valuable insights for future extensions of this important research on schizophrenia and emotion processing.

*Answer: Thank you very much for this valuable information. We will gladly incorporate these methodological tips and insights into our future work.*

Reviewer #2:

The article addresses a relatively unexplored issue: the role of parental attachment in facial emotion recognition (FER) among individuals with schizophrenia in forensic settings. Below some methodological and scientific limitations:

1. The sample size (n = 32) is limited and does not allow for generalizable conclusions. Additionally, one parenting style category (“affectionate constraint”) is not represented in the sample. Please, include a power analysis to justify the sample size or explicitly acknowledge the lack of such analysis as a limitation.

*Answer: This manuscript presents an exploratory analysis of a secondary endpoint of the study published by Hachtel, H. et al. 2024 (MS reference 56), thus an a priori power analysis was conducted, but with respect to the primary outcome of that study and a different design.*

*(Hachtel H, Deuring G, Graf M, Vogel T. Impact of psychosocial stress on facial emotion recognition in schizophrenia and controls: an experimental study in a forensic sample. Front Psychiatry. 2024 Jul 16;15:1358291. doi: 10.3389/fpsyt.2024.1358291. PMID: 39081531; PMCID: PMC11287427.)*

*The commented power analysis itself can be downloaded here (open access): [https://www.frontiersin.org/articles/10.3389/fpsyt.2024.1358291/full#supplementary-material](https://www.frontiersin.org/articles/10.3389/fpsyt.2024.1358291/full#supplementary-material)*

*We additionally emphasized that secondary, exploratory analysis aspect in the manuscript.*

*It is inherently unpredictable how the four-categories PBI parenting style classification will turn out before a study, incidences are skewed among different populations. Our results may serve as a starting point for future studies on the subject.*

*The Generalized Linear Model (GLM) revealed medium to large effect sizes (Cohen's ω_p_² ≥ 0.06) and significant post hoc contrasts exhibited large effect sizes (Hedges’ g ≥ 1.34). When comparing the parameter and contrast estimates from the original analyses to those obtained via bootstrapping, only minimal discrepancies (≤ 3%) were observed, while bootstrapped post hoc contrast effect sizes (Hedges’ g) were estimated ~5% larger. This consistency between analytical and bootstrapped estimates supports our confidence in the reliability of the findings.*

2. Although justified to minimize gender-related biases in FER, this choice significantly limits the generalizability of the findings. Please discuss in more depth the potential gender differences in schizophrenia spectrum disorders and in facial emotion recognition.

*Answer: As we did not have a female group, it is difficult to generalise the results to the other genders. However, most studies have indicated that women tend to perform better at recognising facial emotions, characterised by enhanced accuracy and speed, across a range of emotions and expression intensities. Nevertheless, certain studies have found that this advantage is negligible and not consistently present*.

1. *Wingenbach TSH, Ashwin C, Brosnan M. Sex differences in facial emotion recognition across varying expression intensity levels from videos. Hills PJ, editor. PLoS ONE. 2018 Jan 2;13(1):e0190634.*
2. *Thayer J, Johnsen BH. Sex differences in judgement of facial affect: A multivariate analysis of recognition errors. Scandinavian J Psychology. 2000 Sep;41(3):243–6.*
3. *Kapitanović A, Tokić A, Šimić N. Differences in the recognition of sadness, anger, and fear in facial expressions: the role of the observer and model gender. Archives of Industrial Hygiene and Toxicology. 2022 Dec 30;73(4):308–13.*
4. *Connolly HL, Lefevre CE, Young AW, Lewis GJ. Sex differences in emotion recognition: Evidence for a small overall female superiority on facial disgust. Emotion. 2019 Apr;19(3):455–64.*
5. *Grimshaw GM, Bulman-Fleming MB, Ngo C. A signal-detection analysis of sex differences in the perception of emotional faces. Brain and Cognition. 2004 Apr;54(3):248–50.*
6. *Alotaibi A, Aljuhani A, Alqahtani M, Alahmari N. Sex Differences in Recognition of Face Expressions. Journal of Educational and Psychological Studies. 2023 Oct 3;17(4):384–90.*

3. The use of the PBI, a retrospective self-report instrument, may introduce recall bias or social desirability distortions. to encompass these limits, please cite studies validating the PBI in schizophrenia populations and strengthen the discussion of its methodological limitations.

Thank you for pointing this out. We have included the limitations due to recall bias resulting from a retrospective and self-reported assessment tool in the corresponding section of the manuscript.

*Answer: PBI is a self-reported, retrospective test that may be susceptible to memory bias. The PBI has been shown to demonstrate good internal consistency and construct validity in cases of psychosis and schizophrenia. It has been utilized as a tool to differentiate between clinical and non-clinical groups, with the distinction being based on the experiences of parental bonding reported by the participants. However, PBI is an instrument that is widely used in research for the evaluation of parental attachment, and we are not aware of any more objective instruments for the assessment of parental attachment.*

1. *Roca, M., Vilaregut, A., Palma, C., Barón, F., Campreciós, M., & Mercadal, L. (2020). Basic Family Relations, Parental Bonding, and Dyadic Adjustment in Families with a Member with Psychosis. Community Mental Health Journal, 56, 1262 - 1268.* [*https://doi.org/10.1007/s10597-020-00581-z*](https://doi.org/10.1007/s10597-020-00581-z)*.*
2. *Peh, O., Rapisarda, A., Sim, K., & Lee, J. (2018). T28. QUALITY OF PARENTAL BONDING AMONG INDIVIDUALS WITH ULTRA-HIGH RISK OF PSYCHOSIS AND SCHIZOPHRENIA PATIENTS. Schizophrenia Bulletin, 44, S123 - S124.* [*https://doi.org/10.1093/schbul/sby016.304*](https://doi.org/10.1093/schbul/sby016.304)*.*
3. *Kazarian, S., Baker, B., & Helmes, E. (1987). The Parental Bonding Instrument: factorial structure.. The British journal of clinical psychology, 26 ( Pt 3), 231-2 .* [*https://doi.org/10.1111/J.2044-8260.1987.TB01353.X*](https://doi.org/10.1111/J.2044-8260.1987.TB01353.X)*.*

4. The study does not report on important variables such as current symptomatology (e.g., PANSS), Functioning level (e.g., GAF, PSP) and ongoing pharmacological treatment (type, dosage, duration); please explicitly acknowledge them as potential uncontrolled confounders.

*Answer: The clinical symptoms and psychopathology were evaluated using two scales: the Brief Psychiatric Rating Scale (BPRS) and the Positive and Negative Syndrome Scale (PANSS). The level of psychopathology in the PAT group was low.*

*We have updated the table and included the requested data in the manuscript.*

| Table 1: Demographics and sample characteristics by group. | | | | | | | |
| --- | --- | --- | --- | --- | --- | --- | --- |
|  | Patients | |  | Controls | |  |  |
|  | N=16 | |  | N=16 | |  |  |
|  | Mean | SD |  | Mean | SD | *p** | *IQR* |
| Age (years) | 33.2 | 8.0 |  | 32.8 | 10.6 | .91 |  |
| Education (years) | 11.7 | 2.5 |  | 12.5 | 2.1 | .34 |  |
| Illness duration (years) | 11.6 | 7.9 |  |  |  |  |  |
| PBI mother care | 23.4 | 6.8 |  | 24.1 | 8.3 | .60 |  |
| PBI mother overprotection | 11.4 | 5.0 |  | 11.1 | 7.8 | .36 |  |
| PBI father care | 22.9 ^1^ | 7.0 |  | 21.7 | 7.0 | .65 |  |
| PBI father overprotection | 10.4 ^1^ | 5.2 |  | 8.3 | 3.7 | .21 |  |
| PANSS-positive scale | 12 | 4.0 |  |  |  |  |  |
| PANNS-negative scale | 15.9 | 5.2 |  |  |  |  |  |
| BPRS | 31.5 | 4.5 |  |  |  |  |  |
| Chlorpromazine equivalent (mg) | 618 | 438 |  |  |  |  |  |
| Medication intake (years) | 10 |  |  |  |  |  | 10.08 |
| ^1^ N=14, 2× no report on paternal bonding style; *Student’s t-test or Wilcoxon rank-sum test was applied for group comparisons depending on the data distribution. | | | | | | | |

1. *Overall JE, Gorham DR. The brief psychiatric rating scale. psychol Rep. (1962) 10:199–812. doi: 10.2466/pr0.1962.10.3.799*
2. *Kay SR, Fiszbein A, Opfer LA. The positive and negative syndrome scale (PANSS) for schizophrenia. Schizophr Bull. (1987) 13:261. doi: 10.1093/schbul/13.2.261*

5. Subtest A of the emotional recognition test includes only five basic emotions and a limited number of stimuli. It omits more complex emotions (e.g., contempt, surprise) and variations in intensity. You can richer data incorporating measures such as reaction times and confidence ratings.

*Answer: Using only the five basic emotions is certainly a limitation, however, it is very common in literature that inspired the study procedures. The FER A paradigm was originally embedded in a study protocol of 2 hrs. duration, (see Hachtel et al., 2024), so the limitation was also convenient to keep the number of trials in the experiment at a necessary yet bearable level for the study subjects.*

*Confidence ratings were not taken; this may be an aspect of the paradigm worth improving in future studies. As the focus of the FER A paradigm lies merely on the accurate emotion recognition and not on the recognition speed, we did not analyse that aspect.*

6. Although the authors acknowledge the correlational nature of the study, certain statements (e.g., “optimal parenting could ameliorate...”) may be interpreted as causal or overly speculative. I think that it will be better to reformulate these statements with greater caution, using expressions such as “could be associated with...” or “might mitigate...”.

*Answer: These sections have been corrected and modified.*

Additional suggestions for improving the scientific strength of the paper would be:
1.to include correlations between PBI scores and FER error rates (FERerr) within each group, not just main effects and interactions;

*Answer: We did not find any correlations between the PBI scores and FERerr, however, we added the following sentence to the manuscript:*

*“Spearman’s ρ correlation coefficients between the PBI scores and FERerr, for both groups pooled and each group separate, ranged between absolute 0.07 and 0.36, while none of them was statistically significant (0.72 ≥ p ≥ 0.17; see Supplementary Table 4).”*

*We also added the following table to the Supplementary Material:*

*Supplementary Table 4*: Spearman’s ρ correlation coefficients between the PBI scores and FERerr.

| **CTL & PAT pooled** | **ρ** | ***p*** |
| --- | --- | --- |
| PBI Mother Care | 0.22 | 0.23 |
| PBI Mother Overprotection | -0.19 | 0.30 |
| PBI Father Care | 0.07 | 0.70 |
| PBI Father Overprotection | -0.25 | 0.17 |
|  |  |  |
| **CTL** | **ρ** | ***p*** |
| PBI Mother Care | 0.10 | 0.72 |
| PBI Mother Overprotection | 0.12 | 0.65 |
| PBI Father Care | -0.15 | 0.58 |
| PBI Father Overprotection | 0.21 | 0.45 |
|  |  |  |
| **PAT** | **ρ** | ***p*** |
| PBI Mother Care | 0.10 | 0.71 |
| PBI Mother Overprotection | -0.32 | 0.23 |
| PBI Father Care | 0.36 | 0.21 |
| PBI Father Overprotection | -0.25 | 0.38 |

2. to provide a more detailed discussion of the forensic context: what distinguishes these patients from non-forensic individuals with schizophrenia?

*Answer: A forensic sample contrasts with the population in general psychiatry characterized by reliable diagnostics and monitoring of drug abstinence or medication intake, thereby minimising the risk of medication or substance misuses.*

3. to examine potential differences in FER error patterns—for example, whether certain emotions such as fear are systematically misrecognized, as suggested by previous studies.

*Answer: The evaluation of emotion recognition was based on general error rates. The evaluation of specific emotions, or the error rate in recognising them, was not the focus of the article. In our view, this would be too broad a topic, and the small sample size would not allow for reliable results.*

4. To discuss the role of childhood trauma separately from parental bonding to avoid conceptual confusion between the two constructs.

*Answer: This article did not investigate the influence of childhood trauma on FER. The study focused exclusively on parental attachment. Childhood trauma was addressed in a separate article based on the same study by Hachtel et al. (Front. Psychiatry, 16 July 2024, Sec. Forensic Psychiatry, Vol. 15, 2024,* [*https://doi.org/10.3389/fpsyt.2024.1358291*](https://doi.org/10.3389/fpsyt.2024.1358291)*)*

1. *Hachtel H, Deuring G, Graf M, Vogel T. Impact of psychosocial stress on facial emotion recognition in schizophrenia and controls: an experimental study in a forensic sample. Front Psychiatry. 2024 Jul 16;15:1358291. doi: 10.3389/fpsyt.2024.1358291. PMID: 39081531; PMCID: PMC11287427..*

Overall the article addresses an original and relevant topic in forensic and social psychiatry and is methodologically well-structured. However, it presents several limitations related to sample size, retrospective assessment tools, generalizability, and causal interpretation. With some revisions, this study could make a significant contribution to the understanding of the role of early parental relationships in the social cognition of individuals with schizophrenia, particularly in high-complexity settings such as forensic contexts.

*Answer: Thank you very much for this important comment.*

*We have added the limitations regarding sample size, retrospective assessment tools, and limited generalizability to the manuscript in the appropriate places. The study did not establish causality, but only a statistical correlation. This information has also been added to the manuscript.*

Reviewer #3:

The study explores whether retrospectively assessed parenting style moderates facial emotion recognition (FER) deficits in men with paranoid schizophrenia recruited from Swiss forensic clinics. Sixteen patients and 16 age- and education-matched healthy males completed the Parental Bonding Instrument and a 35-trial FER. A general linear model showed significant main effects of group and parenting style, plus an interaction whereby neglectful parenting was linked to markedly higher FER error rates among patients but not controls. The authors conclude that optimal parental bonding may buffer FER impairment in schizophrenia and suggest clinical and forensic implications. The topic is novel, the paper is clearly organized, and the forensic sample adds ecological relevance for violence-risk research. Nonetheless, several methodological and interpretive issues warrant clarification, as detailed below.
- Prior FER literature is largely cited, but the authors focus exclusively on male forensic patients to control for the potential confounding effects of sex.

*Answer: We had a single-sex patient group to avoid confounding. On the one hand, this is strength of the study, but on the other hand, it is also a weakness, as it limits the generalisability of the study results. Previous studies on facial emotion recognition in women show that, most studies have indicated that women tend to perform better at recognising facial emotions, characterised by enhanced accuracy and speed, across a range of emotions and expression intensities. Nevertheless, certain studies have found that this advantage is negligible and not consistently present. Furthermore, women account for a small minority (typically 4–16%) of forensic psychiatric inpatients across countries. This significantly limits the possibility of recruiting a sample.*

1. *Wingenbach, T., Ashwin, C., & Brosnan, M. (2018). Sex differences in facial emotion recognition across varying expression intensity levels from videos. PLoS ONE, 13.* [*https://doi.org/10.1371/journal.pone.0190634*](https://doi.org/10.1371/journal.pone.0190634)*.*
2. *Thayer, J., & Johnsen, B. (2000). Sex differences in judgement of facial affect: a multivariate analysis of recognition errors.. Scandinavian journal of psychology, 41 3, 243-6 .* [*https://doi.org/10.1111/1467-9450.00193*](https://doi.org/10.1111/1467-9450.00193)*.*
3. *Kapitanović, A., Tokić, A., & Šimić, N. (2022). Differences in the Recognition of Sadness, Anger, and Fear in Facial Expressions: The Role of the Observer and Model Gender. Archives of Industrial Hygiene and Toxicology, 73, 308 - 313.* [*https://doi.org/10.2478/aiht-2022-73-3662*](https://doi.org/10.2478/aiht-2022-73-3662)*.*
4. *Connolly, H., Lefevre, C., Young, A., & Lewis, G. (2019). Sex Differences in Emotion Recognition: Evidence for a Small Overall Female Superiority on Facial Disgust.* Emotion*, 19, 455–464.* [*https://doi.org/10.1037/emo0000446*](https://doi.org/10.1037/emo0000446)*.*
5. *Grimshaw, G., Bulman-Fleming, M., & Ngo, C. (2004). A signal-detection analysis of sex differences in the perception of emotional faces. Brain and Cognition, 54, 248-250.* [*https://doi.org/10.1016/j.bandc.2004.02.029*](https://doi.org/10.1016/j.bandc.2004.02.029)*.*
6. *Alotaibi, A., Aljuhani, A., Alqahtani, M., & Alahmari, N. (2023). Sex Differences in Recognition of Face Expressions. Journal of Educational and Psychological Studies[JEPS].* [*https://doi.org/10.53543/jeps.vol17iss4pp384-390*](https://doi.org/10.53543/jeps.vol17iss4pp384-390)
7. *Streb, J., Lutz, M., Dudeck, M., Klein, V., Maaß, C., Fritz, M., & Franke, I. (2022). Are Women Really Different? Comparison of Men and Women in a Sample of Forensic Psychiatric Inpatients.* Frontiers in Psychiatry*, 13.* [*https://doi.org/10.3389/fpsyt.2022.857468*](https://doi.org/10.3389/fpsyt.2022.857468)*.*
8. *Parfene-Banu, M., Tudorache, M., & Manea, M. (2021). Gender differences in a sample of Romanian forensic psychiatric inpatients.* Romanian Medical Journal*.* [*https://doi.org/10.37897/rmj.2021.4.16*](https://doi.org/10.37897/rmj.2021.4.16)*.*
9. *Felhi, R., Hamdi, G., Chebli, S., & Ridha, R. (2023). Psychiatric and criminological profile of female inpatients in Tunisia.* European Psychiatry*, 66, S884 - S884.* [*https://doi.org/10.1192/j.eurpsy.2023.1871*](https://doi.org/10.1192/j.eurpsy.2023.1871)*.*
10. *Buongiorno, L., Carabellese, F., Margari, A., Parente, L., Mandarelli, G., Catanesi, R., & Carabellese, F. (2024). Sex and gender differences in the Italian forensic psychiatric population: a residences for the execution of security measures study.* International Review of Psychiatry*, 36, 794 - 802. https://doi.org/10.1080/09540261.2024.2378070.*

- Did the authors hypothesize that violent behavior would moderate the relationship between schizophrenia and facial emotion recognition, and if so, could they clarify how this informed the study design?

*Answer: Regarding violent behaviour, we did not develop a hypothesis within the scope of the study, which could have influenced the study design.*

*We merely listed the influence of FER on violent behaviour in the discussion to generate possible research topics/ideas for further studies.*

- Sample size (N = 32) is small; was an a priori power calculation performed?

*Answer: As this manuscript presents an exploratory analysis of a secondary endpoint of the study published by Hachtel, H. et al. 2024 (MS reference 56), the a priori power analysis was conducted with respect to the primary outcome of that study and a different design. The commented power analysis itself can be downloaded here (open access): [https://www.frontiersin.org/articles/10.3389/fpsyt.2024.1358291/full#supplementary-material](https://www.frontiersin.org/articles/10.3389/fpsyt.2024.1358291/full#supplementary-material)*

1. *Hachtel H, Deuring G, Graf M, Vogel T. Impact of psychosocial stress on facial emotion recognition in schizophrenia and controls: an experimental study in a forensic sample. Front Psychiatry. 2024 Jul 16;15:1358291. doi: 10.3389/fpsyt.2024.1358291. PMID: 39081531; PMCID: PMC11287427.*

- Multiple post-hoc contrasts were run—were p-values adjusted (e.g., Bonferroni or Holm) to control Type I error?

*Answer: To clarify the nature of the significant group x parenting style interaction, we performed post hoc contrast analyses of simple main effects. This means testing the levels of one factor within the levels of the second factor against each other. In a 2 x 2 factorial design, the interaction effect has only one degree of freedom. Unlike pairwise testing, simple effects testing represents a decomposition of that single contrast rather than independent multiple comparisons, thus it does not impose an inflation of the family-wise type 1 error rate. Consequently, we did not correct for multiple testing. However, the reported contrasts remain statistically significant even when the p-values are adjusted using Šidák or Tukey corrections*.

- The PBI’s 25-item version is specified, yet cut-offs are provided without citation; …

*Answer: Thank you for pointing out this lack of clarity. We had introduced the section on the PBI with a reference to Parker G, Tupling H, et al. (1979) (reference 58), wherein the parental style scoring schema is described. For better clarity, we added another reference to that original PBI publication before the scoring part in the manuscript.*

*While revising that manuscript segment, we noticed that the cut-off values were not accurately represented; the cut-off value itself was described in the manuscript as being included in the low category, while it really is included in the high category. We corrected the <, >, ≤, and ≥ signs.*

- … would the authors report reliability coefficients (Cronbach’s α) for care/overprotection in this sample?

*Answer: We did not anticipate this request and the validation of the PBI reliability is outside the scope of this work, thus only the aggregated maternal/paternal care/overprotection scores are readily available, but not the item responses. So, we do not report Cronbach’s α.*

- The decision to merge maternal and paternal PBI scores may obscure parent-specific effects; have the authors tested mother vs father bonding separately?

*Answer: Thank you for pointing out this aspect, we added this paragraph to the manuscript:*

*Parent specific effects were not found in our sample, a GLM including parent (father/mother) as additional factor did not show a significant effect for parent, (F_(1,45)_ = 0.099, p = 0.75), while the main effect group and the interaction effect group × parenting style remained unchanged; the marginally significant main effect parenting style was not significant anymore, p = 0.06, due to model complexity.*

- The authors suggest patients with optimal attachment “learn effectively” pre-morbidly; can they cite developmental data supporting this protective mechanism?

*Answer: The social learning behaviour of individuals with schizophrenia undergoes significant changes following the onset of the illness, primarily due to impairments in social cognition, emotion recognition and theory of mind. While premorbid social learning is usually intact, deficits in interpreting social cues and adapting behaviour are common post-illness.*

1. *Brüne, M. (2005). Emotion recognition, ‘theory of mind,’ and social behavior in schizophrenia. Psychiatry Research, 133, 135-147.* [*https://doi.org/10.1016/j.psychres.2004.10.007*](https://doi.org/10.1016/j.psychres.2004.10.007)*.*

2. *Brüne, M., Abdel-Hamid, M., Lehmkämper, C., & Sonntag, C. (2007). Mental state attribution, neurocognitive functioning, and psychopathology: What predicts poor social competence in schizophrenia best?. Schizophrenia Research, 92, 151-159.* [*https://doi.org/10.1016/j.schres.2007.01.006*](https://doi.org/10.1016/j.schres.2007.01.006)*.*

3. *Langdon, R., Connors, M., & Connaughton, E. (2014). Social cognition and social judgment in schizophrenia. Schizophrenia Research: Cognition, 1, 171 - 174.* [*https://doi.org/10.1016/j.scog.2014.10.001*](https://doi.org/10.1016/j.scog.2014.10.001)*.*
4. *Nikolaides, A., Miess, S., Auvera, I., Müller, R., Klosterkötter, J., & Ruhrmann, S. (2016). Restricted attention to social cues in schizophrenia patients. European Archives of Psychiatry and Clinical Neuroscience, 266, 649-661.* [*https://doi.org/10.1007/s00406-016-0705-6*](https://doi.org/10.1007/s00406-016-0705-6)*.*
5. *Merchant, J., Barch, D., Ermel, J., Moran, E., & Butler, P. (2023). Differential deficits in social versus monetary reinforcement learning in schizophrenia: Associations with facial emotion recognition.. Journal of psychopathology and clinical science.* [*https://doi.org/10.1037/abn0000869*](https://doi.org/10.1037/abn0000869)*.*

- Clinical implications mention neuromodulation studies but do not connect directly to parenting interventions; I am curious about how realistic the proposal is to modify parental bonding retrospectively in adult populations.

*Answer: Existing therapeutic approaches using neuromodulation showed remarkable effects not on attachment style retrospectively, but on recognizing of facial emotions, which helps to improve the social skills.*

1. *Yamada Y, Inagawa T, Hirabayashi N, Sumiyoshi T. Emotion Recognition Deficits in Psychiatric Disorders as a Target of Non-invasive Neuromodulation: A Systematic Review. Clin EEG Neurosci. 2022 Nov;53(6):506–12.*

- The affectionless control group was excluded due to imbalance—could an exploratory analysis retaining this category (with caution) be placed in Supplementary Material?

*Answer: The cell CTL-Affectionless control holds only 4 cases, so we excluded this parenting style, because N = 4 is really too small for a meaningful analysis and we don’t think it would yield any reliable information.*

- Occasional grammatical slips (e.g., “was has been approved,” p.6) need correction.

*Answer: The grammatical error has been removed.*
